# Biological subtype predicts locoregional recurrence after postmastectomy radiotherapy in Chinese breast cancer patients

**DOI:** 10.1002/cam4.2904

**Published:** 2020-02-12

**Authors:** Jiangfeng Wang, Jurui Luo, Kairui Jin, Xuanyi Wang, Zhaozhi Yang, Jinli Ma, Xin Mei, Xiaofang Wang, Zhirui Zhou, Xiaoli Yu, Xingxing Chen, Xiaomao Guo

**Affiliations:** ^1^ Department of Radiation Oncology Fudan University Shanghai Cancer Center Shanghai China; ^2^ Department of Oncology Shanghai Medical College Fudan University Shanghai China

**Keywords:** biological subtype, breast cancer, radiotherapy, recurrence

## Abstract

**Aim:**

To investigate the impact of biological subtypes in locoregional recurrence in Chinese breast cancer patients receiving postmastectomy radiotherapy (PMRT).

**Methods and Materials:**

About 583 patients who received postmastectomy radiation between 2010 and 2012 were retrospectively analyzed. According to immunohistochemical staining profile, patients were classified into: Luminal A‐like, Luminal B‐like, HER2‐positive, and triple‐negative breast cancer (TNBC). Local and regional recurrence (LRR) cumulative incidences were calculated by competing risks methodology and the power of prognostic factors was examined by Gray's test and the test of Fine and Gray.

**Results:**

The median follow‐up was 70.9 months. About 34 LRR events occurred. For Luminal A, Luminal B, HER2‐positive, and TNBC patients, the 5‐year LRR cumulative incidence rates were 1.57%, 4.09%, 10.74%, and 10.28%. Compared with Luminal A, HER2‐positive subtype and TNBC had a significant increased risk of LRR (HR was 5.034 and 5.188, respectively). In univariate analysis, predictive factors for higher LRR were HER2‐positive subtype (HR = 4.43, *P* < .05), TNBC (HR = 4.70, *P* < .05), and pN3 (HR = 5.83, *P* < .05). In the multivariate model, HER2‐positive subtype (HR = 5.034, *P* < .05), TNBC (HR = 5.188, *P* < .05), and pN3 (HR = 9.607, *P* < .01) were independent predictors of LRR. LRR without trastuzumab was similar to that of TNBC (without vs TNBC, 17.88% vs 10.28%, *P* > .05) in HER2‐positive subtype patients, while LRR with trastuzumab was approximate to Luminal A (with vs Luminal A, *P* > .05). Additionally, endocrine therapy also significantly reduced LRR incidence in the luminal subtype cohort (without vs with therapy, 6.25% vs 2.89%, HR = 0.365, *P* < .1).

**Conclusions:**

Biological subtype was a prognostic factor of LRR in the PMRT setting among Chinese breast cancer patients.

## Graphical abstract

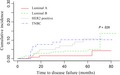

## 1 INTRODUCTION

A majority of breast cancer patients undergoing mastectomy will receive radiotherapy as a local treatment, which will reduce local and regional recurrence (LRR), further elevating survival.^1^ But there is still 10% breast cancer patients who received postmastectomy radiotherapy (PMRT) suffered from local recurrence, indicating undertreatment for these patients.^2^ Therefore, the identification of patients who are at higher risk of recurrence and need more aggressive treatment is imperative.

Several multigene molecular assays are currently under investigation to accurately identify patients with unfavorable prognosis.^3^, ^4^ However, considering the lack of cost‐effectiveness of these detection approaches, they are not easily applied on a large scale, especially in less developed area and countries. In contrast, immunohistochemical (IHC) staining profile is a cost‐effective and popular surrogate.^5^ Based on IHC staining profiles, St Gallen International Breast Cancer Conference in 2013 classified breast cancer into four subtypes: Luminal A, Luminal B, HER2‐positive, and triple‐negative breast cancer (TNBC). This classification system has been widely used in clinical decision‐making for the systemic management of breast cancer.^6^

There were few studies to investigate the relationship between biological subtype and locoregional recurrence after PMRT. Wright et al analyzed 582 consecutively treated patients receiving PMRT and demonstrated that biological subtype could predict the prognosis of different biological subtypes of breast cancer.^7^ Notably, this study was limited in only examining specific ethic groups (black and white patient groups). However, racial disparities between Chinese and Western patients are remarkably huge in regard to gene profiling, recurrence pattern, and distribution of molecular subtypes. For example, one study from our center compared somatic mutation frequencies of Chinese breast cancer patients with those in the TCGA dataset and found that compared with Western patients, Chinese breast cancer patients have higher frequencies of PIK3R1 somatic mutation, which contribute to cancer development and drug resistance, irrespective of biological subtype.^8^ In addition, Yin et al reported double‐peaked time distribution of recurrence risk in Chinese breast cancer patients, which was different from the single recurrence risk peak reported by a study in a Western population.^9^ These results addressed the potential genetic and clinical differences between Chinese and Western cohorts and thus highlighted the need for studies that focused on Chinese cohorts to personalize local treatment.

Whether biological subtype can serve as a prognostic biomarker for patients who received PMRT has been seldom examined in a Chinese cohort. Therefore, we conducted the present analysis on the relationship between LRR and biological subtypes of breast cancer patients undergoing PMRT in Fudan University Shanghai Cancer Center in order to provide more insights into clinical decision.

## 2 METHOD

### 2.1 Patients

From January 2010 to July 2012, 688 patients who treated with mastectomy and PMRT at the Department of Radiation Oncology, Fudan University Shanghai Cancer Center were retrospectively reviewed. Their medical records were consecutively collected. Review of data for this investigation was approved by the Institutional Review Board of our center. Before treatment, all patients were required to have a complete physical examination, routine blood test, biochemical test, mammography and/or breast ultrasonography, chest X‐ray, an abdominopelvic computed tomography scan and/or ultrasonography, and a bone scan if indicated. The 2018 eighth edition of the AJCC Cancer Staging Manual was used for restaging.^10^

The inclusion criteria were as follows^1^: pathologically confirmed invasive breast cancer^2^; both mastectomy and radiotherapy were performed in our center; and^3^ available and complete information of IHC stainings for estrogen receptor (ER), progesterone receptor (PR), and Ki‐67 and IHC stainings and fluorescence in situ hybridization (FISH) analyses for HER2 receptor (HER2). Patients with distant metastasis at the time of treatment were excluded from the present investigation. A total of 583 patients fulfilled the inclusion criteria and were included in the present study.

### 2.2 Treatment

All patients received mastectomy and axillary dissection. Radiotherapy and/or chemotherapy was performed according to the National Comprehensive Cancer Network practice guideline. PMRT was compulsory for patients with T3‐T4 and/or N2‐N3 breast cancer, while elective for the T1‐T2N1 cohort. With a 6‐MV linear accelerator, radiotherapy was delivered to the ipsilateral chest wall, supraclavicular region of lymph nodes, and/or intramammary lymph nodes. The total dose was 50 Gy /25 fractions. Nine patients received an additional 10 Gy to the tumor bed.

### 2.3 Biological subtypes

ER, PR, and Ki‐67 were assessed by IHC staining. IHC staining of ER, PR, and Ki‐67, more than 1%, 20%, and 20%, respectively, was considered positive or high expression.^6^ HER2 was considered positive with a staining score over 3. If the staining for HER2 was intermediate or the score was 2, FISH analysis was conducted, with HER2 gene amplification considered positive.

Tumors were subtyped according to the St Gallen International Breast Cancer Conference (2013) Expert Panel as Luminal A‐like (ER‐positive, PR‐positive, HER2‐negative, and low Ki‐67), Luminal B‐like (ER‐positive, HER2‐negative, and at least one of: high Ki‐67 or negative or low PR; ER‐positive, HER2 overexpressed or amplified, any Ki‐67 and any PR), HER2‐positive (HER2 overexpressed or amplified‐ER and PR absent), and TNBC (ER‐negative, PR‐negative, HER2‐negative, and any Ki‐67).^6^

### 2.4 Follow‐up

Follow‐up was determined from the date of diagnosis. Outpatient department records and regular telephone calls were conducted to perform follow‐up. Information from personal contact with the patients was also collected. LRR diagnosis was defined by pathology or image information (such as CT, MRI, or ultrasonography). The date of LRR was the date of the first relapse that occurred in the ipsilateral chest wall, supraclavicular lymph nodes, axillary nodes, or the intramammary chain.

### 2.5 Statistical analysis

LRR rate was analyzed as the first site of failure. Cumulative incidence of LRR was calculated by competing risks data analysis, as described by Gray,^11^ with distant metastasis alone and deaths as competing risks. Gray's test was used to compare cumulative incidence curves. Grade, lymphovascular invasion (LVI), tumor size, lymph node involvement, and subtype classification were included and analyzed by univariate analysis and multivariate analysis with the Fine and Gray's competing risk regression model.^12^ The “cuminc” and “crr” procedure in the R statistical package “cmprsk” were applied. Annual LRR hazard rates were estimated with a Kernel method of smoothing. SPSS version 25.0 (SPSS, Chicago, IL, USA) and R software version 2.11.1 were used for all statistical analyses.

## 3 RESULTS

### 3.1 Patient characteristics

Clinicopathological factors of all patients and the four subgroups are shown in Table 1. According to the IHC results for molecules, 127 patients (21.8%), 275 patients (47.2%), 103 patients (17.7%), and 78 patients (13.4%) were Luminal A, Luminal B, HER2‐positive, and TNBC, respectively. The median age of the patient group at diagnosis was 50 years (range, 25‐76 years). The T stage for most of the patients (494, 84.7%) was T1‐2. A total of 77 (13.2%) patients were negative for lymph node involvement; 220 patients (37.7%) had 1‐3 nodes involved, 148 (25.4%) had 4‐9 nodes, and 138 (23.7%) had more than 10. All patients underwent mastectomy and radiotherapy. Chemotherapy was received among 94.0% patients. Among HER2‐positive patients, 53.4% received trastuzumab‐containing therapy. Endocrine treatment was administered to almost all patients (88.1%) who were ER‐ or PR‐positive. All patients received radiation to the chest wall and/or regional lymph nodes, and the median dose inclusive of boost was 50 Gy (range 40‐64). About 61.76% of recurrent patients were diagnosed by both image and pathology. Others were diagnosed by image.

**Table 1:** Clinical characteristics per subtype and treatment of 583 patients

	All (n = 583)	Luminal A (n = 127, 21.8%)	Luminal B (n = 275, 47.2%)	HER2‐positive (n = 103, 17.7%)	TNBC (n = 78, 13.4%)
Age at diagnosis >50 y, No. (%)	277 (47.5%)	54 (42.5%)	132 (48%)	54 (52.4%)	37 (47.3%)
Median (minimum, maximum)	50 (25, 76)	49 (28, 72)	50 (27, 76)	52 (25, 71)	49 (27, 72)
Menopausal status, No. (%)					
Pre‐	289 (49.6%)	71 (55.9%)	137 (49.8%)	48 (46.6%)	33 (42.3%)
Post‐	273 (46.8%)	54 (42.5%)	129 (46.9%)	50 (48.5%)	40 (51.3%)
Unknown	21 (3.6%)	2 (1.6%)	9 (3.3%)	5 (4.9%)	5 (6.4%)
Pathology, No. (%)					
IDC	570 (97.8%)	123 (96.9%)	268 (97.5%)	102 (99.0%)	77 (98.7%)
ILC	12 (2.1%)	4 (3.1%)	6 (2.2%)	1 (1.0%)	1 (1.3%)
Other	1 (0.2%)	0 (0)	1 (0.4)	0 (0%)	0 (0)
Grade, No. (%)					
1‐2	297 (50.9%)	92 (72.4%)	150 (54.5%)	36 (35.0%)	19 (24.4%)
3	167 (28.6%)	23 (18.1%)	74 (26.9%)	34 (33.0%)	36 (46.2%)
Unknown	119 (20.4%)	12 (9.4%)	51 (18.5%)	33 (32.0%)	23 (29.5%)
LVI, No. (%)					
Yes	375 (64.3%)	97 (76.4%)	179 (65.1%)	57 (55.3%)	42 (53.8%)
No	163 (28.0%)	27 (21.3%)	80 (29.1%)	29 (28.2%)	27 (34.6%)
Unknown	45 (7.7%)	3 (2.4%)	16 (5.8%)	17 (16.5%)	9 (11.5%)
T stage, No. (%)					
T1‐2	494 (84.7%)	122 (96.1%)	236 (85.8%)	80 (77.7%)	56 (71.8%)
T3	23 (3.9%)	2 (1.6%)	12 (4.4%)	5 (4.9%)	4 (5.1%)
Tx	66 (11.3%)	3 (2.4%)	27 (9.8%)	18 (17.5%)	18 (23.1%)
N stage, No. (%)					
N0	77 (13.2%)	7 (5.5%)	29 (10.5%)	24 (23.3%)	17 (21.8%)
N1	220 (37.7%)	47 (37.0%)	108 (39.3%)	36 (35.0%)	29 (37.2%)
N2	148 (25.4%)	38 (30.0%)	76 (27.6%)	21 (20.4%)	13 (16.7%)
N3	138 (23.7%)	35 (27.6%)	62 (22.5%)	22 (21.4%)	19 (24.4%)
Breast surgery, No. (%)					
Mastectomy	583 (100%)	127 (100%)	275 (100%)	103 (100%)	78 (100%)
Radiotherapy, No. (%)					
Yes	583 (100%)	127 (100%)	275 (100%)	103 (100%)	78 (100%)
Chemotherapy, No. (%)					
Yes	548 (94.0%)	118 (92.9%)	259 (94.2%)	96 (93.2%)	75 (96.2%)
No	35 (6%)	9 (7.1%)	16 (5.8%)	7 (6.8%)	3 (3.8%)
Hormone therapy, No. (%)					
Yes	392 (67.2%)	116 (91.3%)	238 (86.5%)	34 (33.0%)	4 (5.1%)
No	191 (32.8%)	11 (8.7%)	37 (13.5%)	69 (67.0%)	74 (94.9%)
Trastuzumab, No. (%)					
Yes	114 (19.6%)	1 (0.8%)	58 (21.1%)	55 (53.4%)	0 (0)
No	469 (80.4%)	126 (99.2%)	217 (78.9%)	48 (46.6%)	78 (100%)

Abbreviations: IDC, invasive ductal carcinoma; ILC, invasive lobular carcinoma; LVI, lymphatic vessel invasion; TNBC, triple‐negative breast cancer

### 3.2 Patterns and hazard rate of LRR

**Figure 1:**
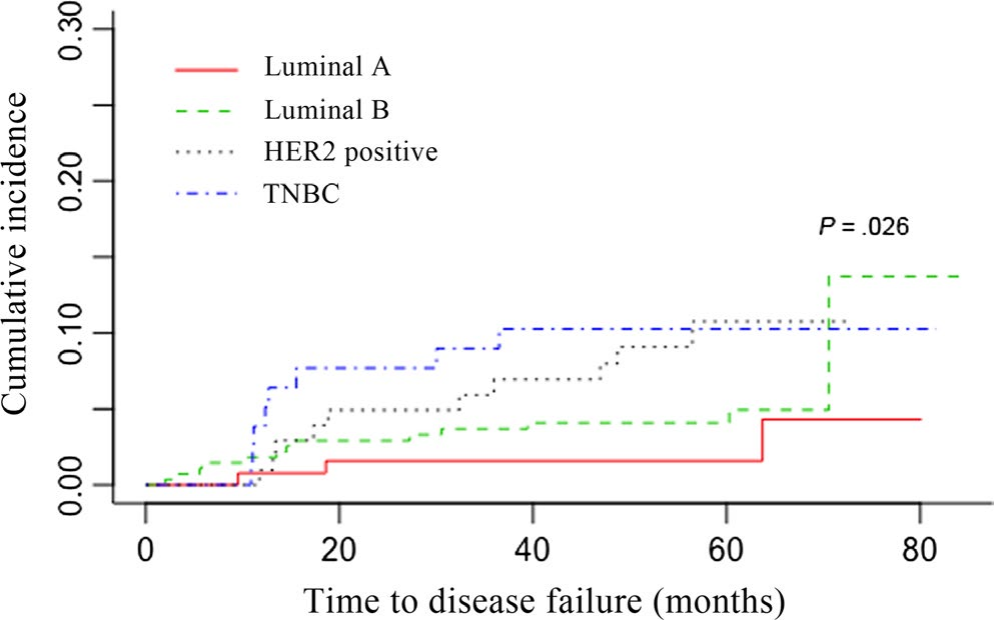
Cumulative incidence rate of LRR as first failure within different subtypes

**Figure 2:**
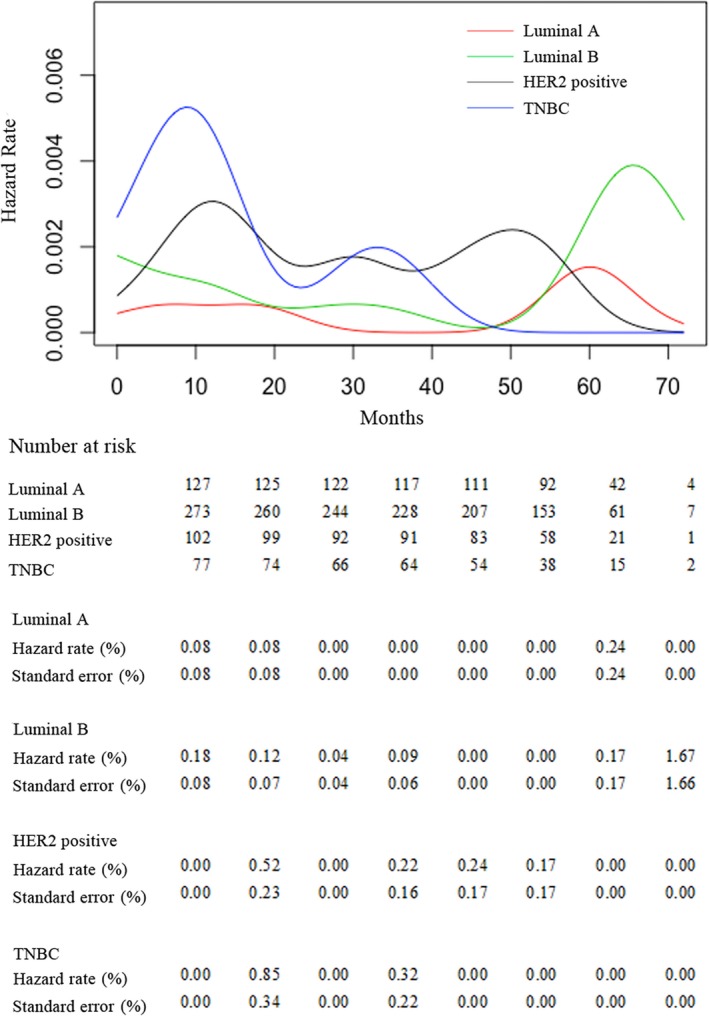
Locoregional relapse hazard rates for different subgroups in 583 Chinese breast cancer patients

With a median follow‐up time of 70.9 months, a total of 34 patients developed LRR. For the entire cohort, the 5‐year cumulative incidence of LRR (± synchronous distant metastasis) was 5.54%. Twenty‐eight patients were diagnosed of LRR concurrently with distant failure and six patients were diagnosed with isolated LRR. Among the 34 patients who developed LRR, there were 39 sites of recurrence, including supraclavicular area (n = 16), chest wall (n = 15), internal mammary area (n = 4), and axillary nodes (n = 4) (Figures S1 and S2). For Luminal A, Luminal B, HER2‐positive, and TNBC patients, the 5‐year LRR cumulative incidence rates were 1.57%, 4.09%, 10.74%, and 10.28%, respectively (Figure 1 and Table 2). In terms of LRR time, all LRR occurred in the first 3 years in the TNBC subtype, while for HER2‐positive subtype, 90% LRR occurred in the first 4 years and only one LRR occurred in the fifth year. In contrast, after the fifth year of follow‐up, the luminal subtype population had a 18.75% LRR rate. This was also revealed in the analysis of hazard function showing earlier relapse peak in TNBC and later in luminal subtypes (Figure 2).

**Table 2:** Cumulative rates for LRR (with and without DM) by molecular subtypes after every 20‐months follow‐up

	Luminal A	Luminal B	HER2‐positive	TNBC
20‐mo^a^	1.57%	2.92%	4.92%	7.69%
40‐mo^a^	1.57%	4.09%	6.94%	10.28%
60‐mo^a^	1.57%	4.09%	10.74%	10.28%
80‐mo^a^	4.32%	13.73%	10.74%	10.28%
*P* value^b^	.026			

Note

Isolated distant metastasis and death as competing risks.

Abbreviations: DM, distant metastasis; TNBC, triple‐negative breast cancer.

a 60‐mo cumulative incidence rate in percentage.

b Gray's test was used to compare cumulative incidence curves.

### 3.3 Prognostic factors associated with LRR

Factors predictive of LRR were analyzed by univariate analysis and multivariate analysis. In univariate analysis, factors predictive of higher LRR were HER2‐positive subtype (HR = 4.43, 95% CI 1.234‐15.93, *P* < .05), TNBC (HR = 4.70, 95% CI 1.251‐17.63, *P* < .05), and pN3 (HR = 5.83, 95% CI 1.356‐25.05, *P* < .05). Grade, pT, and LVI statuses were not related to LRR (Table 3). As shown in the multivariate model (Table 4), HER2‐positive subtype (HR = 5.034, 95% CI 1.357‐18.67, *P* < .05), TNBC (HR = 5.188; 95% CI 1.335‐20.16, *P* < .05), and pN3 (HR = 9.607, 95% CI 2.053‐44.96, *P* < .01) were independent predictors of LRR. HER2‐positive and TNBC had increased risk of LRR, while Luminal B had a nonsignificant higher risk than Luminal A.

**Table 3:** Univariate analysis for locoregional recurrence‐free survival

	HR	95% confidence interval		*P* value^a^
		Lower	Upper	
Subtype				
Luminal A	1			
Luminal B	2.12	0.606	7.39	.240
HER2‐positive	4.43	1.234	15.93	.022
TNBC	4.70	1.251	17.63	.022
Grade				
1‐2	1			
3	1.25	0.612	2.57	.54
pT				
1	1			
2	0.856	0.425	1.72	.66
3	1.961	0.512	7.51	.33
pN				
0	1			
1	1.24	0.257	5.98	.790
2	1.59	0.323	7.87	.570
3	5.83	1.356	25.05	.018
LVI				
0	1			
1	1.32	0.63	2.76	.46

Note

Isolated distant metastasis and death as competing risks.

Abbreviations: TNBC, triple‐negative breast cancer; LVI, lymphatic vessel invasion.

a Significance of hazard ratio (HR) was calculated with Fine and Gray's competing risk regression model.

**Table 4:** Multivariate analysis for locoregional recurrence‐free survival

	HR	95% confidence interval		*P* value^a^
		Lower	Upper	
Subtype				
Luminal A	1			
Luminal B	2.313	0.665	8.04	.1900
HER2‐positive	5.034	1.357	18.67	.0160
TNBC	5.188	1.335	20.16	.0170
Grade				
1‐2	1			
3	0.899	0.396	2.04	.8000
pT				
1	1			
2	0.713	0.334	1.52	.3800
3	1.785	0.511	6.24	.3600
pN				
0	1			
1	1.947	0.363	10.45	.4400
2	2.654	0.514	13.72	.2400
3	9.607	2.053	44.96	.0041
LVI				
0	1			
1	0.993	0.443	2.22	.9900

Note

Isolated distant metastasis and death as competing risks.

Abbreviations: TNBC, triple‐negative breast cancer; LVI, lymphatic vessel invasion.

a Significance of hazard ratio (HR) was calculated with Fine and Gray's competing risk regression model.

### 3.4 Predictive value of trastuzumab

**Figure 3:**
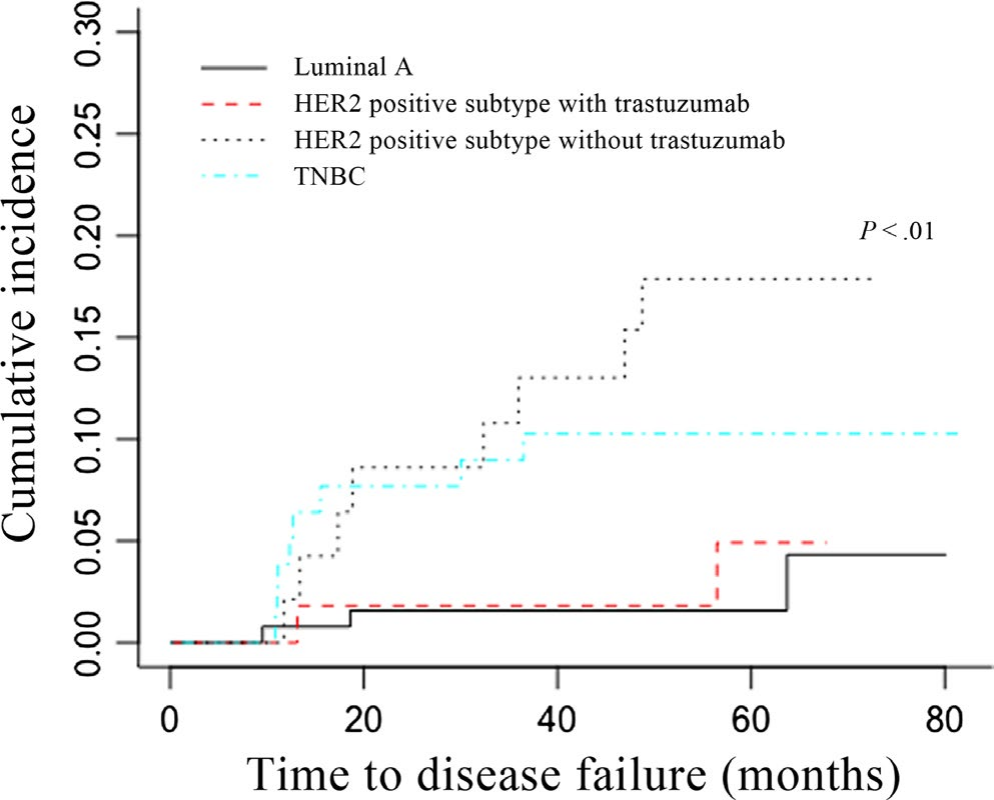
Cumulative incidence rate of LRR as first failure within Luminal A, TNBC, and HER2‐positive with and without target treatment. TNBC, Triple‐negative breast cancer

In the present study, the LRR incidence for HER2‐positive and ‐negative was at a similarly low level (7.89% vs 9.71%, *P* > .05). This was not consistent with the report of the Danish Breast Cancer Cooperative Group and the report from Wang and his colleagues^13^, ^14^ showing that HER2‐positive predicted a higher risk of LRR. We hypothesized that this difference may be attributed to the application of HER2‐targeted treatment. Therefore, we analyzed LRR in subgroups of HER2‐positive patients with and without trastuzumab. The results are shown in Table 5 and Figure 3. Compared with the subgroup that did not receive targeted treatment, the trastuzumab group was associated with lower LRR risk (without vs with, 17.88% vs 4.91%, HR = 0.194, *P* < .05). Interestingly, the LRR incidence for HER2‐positive with trastuzumab was similar to that of Luminal A (HR = 1.8, *P* > .05, Luminal A as reference). In addition, the LRR incidence for HER2‐positive without trastuzumab was similar to that of TNBC (HR = 1.66, *P* > .05, TNBC as reference). In addition to trastuzumab, endocrine therapy also significantly reduced LRR incidence (without vs with therapy, 6.25% vs 2.89%, HR = 0.365, *P* < .1).

**Table 5:** 60‐month cumulative incidence rate of LRR among Luminal A, TNBC, HER2‐positive subtype with and without trastuzumab

	Rate^a^	*P* value^b^
Luminal A	1.57%	.00275
HER2‐positive subtype with trastuzumab	4.91%	
HER2‐positive subtype without trastuzumab	17.88%	
TNBC	10.28%	

Note

Isolated distant metastasis and death as competing risks

a 60‐mo cumulative incidence rate in percentage

b Gray's test was used to compare cumulative incidence curves.

## 4 DISCUSSION

In the era of precision medicine, the accurate prediction of patient's prognosis is critical to avoid overtreatment or undertreatment.^15^ The present study demonstrated that molecular subtype based on definitions reported in 2013 St Gallen conference was an independent prognostic predictor for patients who received PMRT and its prognostic value even surpassed that of traditional prognostic factors, including tumor size, tumor grade, and LVI. Moreover, endocrinotherapy and targeted therapy significantly reduced LRR. To the best of our knowledge, the present study is the first to report the prognostic value of biological subtype as defined in the 2013 St Gallen conference in Chinese breast cancer patients who underwent PMRT.

The number of studies addressing the relationship between biological subtype and LRR after mastectomy has been quite limited. For example, Wang et al enrolled 835 node‐positive breast cancer patients with mastectomy and reported that patients with triple‐negative and HER2 + expression profiles had a significantly higher 5‐year LRR rate than those with positive hormone receptors and negative HER2 profiles.^13^ Yolanda et al observed that TNBC patients had the highest risk of LRR compared with other biological subtypes.^16^ Notably, the percentages of patients who received PMRT were only 50.3% and 30% in the studies of Yu et al and Yolanda et al, respectively. To our knowledge, until now, the study cohort published were all from Western countries. The prognostic value of biological subtype for LRR among Chinese breast cancer patients in the setting of PMRT had not been fully characterized.

The definition of molecular subtypes of breast cancer has evolved gradually and steadily according to the better understanding of the biological behavior of breast cancer. After 2013 St Gallen conference, Ki‐67 has been regarded as an important parameter for treatment decision.^6^ We hypothesized the definitions of subtypes including Ki‐67 index may outperform the previous classification without Ki‐67 value in predicting LRR for breast cancer undergo PMRT. The present results confirmed our hypothesis and showed that biological subtype, including Ki‐67 index, predicts locoregional recurrence after PMRT. Therefore, definitions set up by 2013 St Gallen conference is a better prognostic panel of LRR for breast cancer after PMRT in Chinese cohort. We also tried to classify patients according to hormone receptor and HER2 status without Ki‐67 index as other researches.^17^ However, no relationship between biological subtypes and LRR was observed (data not shown).

We also observed remarkably different patterns of relapse among the subtypes. Despite the highest risk of LRR in TNBC, the LRR curve for TNBC demonstrated the steepest incline before the peak, indicating earlier local relapse compared with HER2‐positive or luminal subtypes. In the TNBC cohort, all local recurrence occurred within the first 3 years, which was 1 to 3 years earlier than HER2‐positive and luminal subtypes. A similar relapse pattern was also observed in other studies. Dent et al examined a cohort of 1601 breast cancer patients and analyzed timing of relapse among different biological subtypes. TNBC patients had an earlier local recurrence than other subtypes (2.8 vs 4.2 years, respectively; *P* = .02).^18^ Meena also reported that local relapses in TNBC patients typically happened within the first 5 years, which was much earlier than the luminal subtypes (between 5 and 15 or more years).^19^ Taken together, these results suggest that for TNBC patients, regular and mandatory follow‐up in the first 3 to 5 years and more aggressive and effective local treatment is of great importance. In term of anatomical distribution of LRRs, the present study showed that a greater proportion of LRR was in the ipsilateral chest wall and supraclavicular region. Therefore, chest wall and supraclavicular region should be followed closely. However, considering relatively small sample size from single institution, further investigations are greatly needed.

Overexpression of HER2, one of the most important molecular markers for breast cancer, is closely related to proliferation, invasiveness, and radioresistance of breast cancer cells. Therefore, HER2 overexpression is a negative prognostic predictor in breast cancer patients. In 2008, the FDA approved the clinical application of trastuzumab, which is a humanized monoclonal antibody directed against HER2.^20^ Trastuzumab was shown to improve survival for breast cancer patients with HER2 overexpression, especially when combined with standard chemotherapy or neoadjuvant chemotherapy,^21^, ^22^ suggesting that the prognosis of breast cancer patients could be elevated with the aid of appropriate treatment. In contrast, TNBC remained as the most aggressive subtype of breast cancer due to the lack of treatment target. Fortunately, several clinical trials have shown promising results for TNBC, such as therapies targeting PD‐1 or PARP.^23^, ^24^

There are some inherent limitations in this retrospective observational investigation that should be considered. Firstly, some patients were excluded from this study because complete information of IHC staining for ER, PR, HER2, or Ki‐67 was missing. Therefore, the sample size of the present study was relatively small with 583 included patients. Second, all the patients included were from the same cancer center, which may bring bias to the current study. The results should be further tested in multicenter studies. Third, the duration of the follow‐up was short to some extent. To fully compare the LRR rate among the four subtypes, longer follow‐up is needed.

## 5 CONCLUSION

Our study demonstrated that biological subtype based on the classification standard from the St Gallen International Breast Cancer Conference (2013) Expert Panel serves as a reliable prognostic predictor for Chinese breast cancer patients receiving PMRT. Treatment with trastuzumab and endocrine therapy dramatically decreased the risk of LRR in HER2‐positive and hormonal receptor‐positive patients.

## Data Availability

The data that support the findings of this study are available from the corresponding author upon reasonable request.

## References

[1] Ragaz J , Olivotto IA , Spinelli JJ , et al. Locoregional radiation therapy in patients with high‐risk breast cancer receiving adjuvant chemotherapy: 20‐year results of the British Columbia randomized trial. J Natl Cancer Inst. 2005;97(2):116‐126. 15657341 10.1093/jnci/djh297

[2] Clarke M , Collins R , Darby S , et al. Effects of radiotherapy and of differences in the extent of surgery for early breast cancer on local recurrence and 15‐year survival: an overview of the randomised trials. Lancet. 2005;366(9503):2087‐2106. 16360786 10.1016/S0140-6736(05)67887-7

[3] Torres‐Roca JF , Fulp WJ , Caudell JJ , et al. Integration of a radiosensitivity molecular signature into the assessment of local recurrence risk in breast cancer. Int J Radiat Oncol Biol Phys. 2015;93(3):631‐638. 26461005 10.1016/j.ijrobp.2015.06.021 PMC5811194

[4] Gee HE , Buffa FM , Harris AL , et al. MicroRNA‐related DNA repair/cell‐cycle genes independently associated with relapse after radiation therapy for early breast cancer. Int J Radiat Oncol Biol Phys. 2015;93(5):1104‐1114. 26581147 10.1016/j.ijrobp.2015.08.046

[5] Nielsen TO , Hsu FD , Jensen K , et al. Immunohistochemical and clinical characterization of the basal‐like subtype of invasive breast carcinoma. Clinic Cancer Res. 2004;10(16):5367‐5374. 10.1158/1078-0432.CCR-04-0220 15328174

[6] Goldhirsch A , Winer EP , Coates AS , et al. Personalizing the treatment of women with early breast cancer: highlights of the St Gallen International Expert Consensus on the Primary Therapy of Early Breast Cancer 2013. Annals Oncol. 2013;24(9):2206‐2223. 10.1093/annonc/mdt303 PMC3755334 23917950

[7] Panoff JE , Hurley J , Takita C , et al. Risk of locoregional recurrence by receptor status in breast cancer patients receiving modern systemic therapy and post‐mastectomy radiation. Breast Cancer Res Treat. 2011;128(3):899‐906. 21475999 10.1007/s10549-011-1495-1

[8] Chen LI , Yang L , Yao L , et al. Characterization of PIK3CA and PIK3R1 somatic mutations in Chinese breast cancer patients. Nat Commun. 2018;9(1):1357. 29636477 10.1038/s41467-018-03867-9 PMC5893593

[9] Yin W‐J , Lu J‐S , Di G‐H , et al. Clinicopathological features of the triple‐negative tumors in Chinese breast cancer patients. Breast Cancer Res Treat. 2009;115(2):325‐333. 18563552 10.1007/s10549-008-0096-0

[10] Giuliano AE , Connolly JL , Edge SB , et al. Breast Cancer‐Major changes in the American Joint Committee on Cancer eighth edition cancer staging manual. Cancer J Clinic. 2017;67(4):290‐303. 10.3322/caac.21393 28294295

[11] Gray RJ . A class of K‐sample tests for comparing the cumulative incidence of a competing risk. Annals Statistics. 1988;16(3):1141‐1154.

[12] Kim HT . Cumulative incidence in competing risks data and competing risks regression analysis. Clinic Cancer Res. 2007;13(2 Pt 1):559‐565. 10.1158/1078-0432.CCR-06-1210 17255278

[13] Wang S‐L , Li Y‐X , Song Y‐W , et al. Triple‐negative or HER2‐positive status predicts higher rates of locoregional recurrence in node‐positive breast cancer patients after mastectomy. Int J Radiat Oncol Biol Phys. 2011;80(4):1095‐1101. 20638197 10.1016/j.ijrobp.2010.03.038

[14] Kyndi M , Sorensen FB , Knudsen H , Overgaard M , Nielsen HM , Overgaard J . Estrogen receptor, progesterone receptor, HER‐2, and response to postmastectomy radiotherapy in high‐risk breast cancer: the Danish Breast Cancer Cooperative Group. J Clinic Oncol. 2008;26(9):1419‐1426. 10.1200/JCO.2007.14.5565 18285604

[15] Katz SJ , Morrow M . Addressing overtreatment in breast cancer: the doctors' dilemma. Cancer. 2013;119(20):3584‐3588. 23913512 10.1002/cncr.28260

[16] Tseng YD , Uno H , Hughes ME , et al. Biological subtype predicts risk of locoregional recurrence after mastectomy and impact of postmastectomy radiation in a large national database. Int J Radiat Oncol Biol Phys. 2015;93(3):622‐630. 26461004 10.1016/j.ijrobp.2015.07.006

[17] Lowery AJ , Kell MR , Glynn RW , Kerin MJ , Sweeney KJ . Locoregional recurrence after breast cancer surgery: a systematic review by receptor phenotype. Breast Cancer Res Treat. 2012;133(3):831‐841. 22147079 10.1007/s10549-011-1891-6

[18] Dent R , Trudeau M , Pritchard KI , et al. Triple‐negative breast cancer: clinical features and patterns of recurrence. Clinic Cancer Res. 2007;13(15 Pt 1):4429‐4434. 10.1158/1078-0432.CCR-06-3045 17671126

[19] Moran MS . Radiation therapy in the locoregional treatment of triple‐negative breast cancer. Lancet Oncol. 2015;16(3):e113‐e122. 25752562 10.1016/S1470-2045(14)71104-0

[20] Nemeth BT , Varga ZV , Wu WJ , Pacher P . Trastuzumab cardiotoxicity: from clinical trials to experimental studies. Br J Pharmacol. 2017;174(21):3727‐3748. 27714776 10.1111/bph.13643 PMC5647179

[21] Romond EH , Perez EA , Bryant J , et al. Trastuzumab plus adjuvant chemotherapy for operable HER2‐positive breast cancer. New Engl J Med. 2005;353(16):1673‐1684. 16236738 10.1056/NEJMoa052122

[22] Smith I , Procter M , Gelber RD , et al. 2‐year follow‐up of trastuzumab after adjuvant chemotherapy in HER2‐positive breast cancer: a randomised controlled trial. Lancet (London, England). 2007;369(9555):29‐36. 10.1016/S0140-6736(07)60028-2 17208639

[23] Schmid P , Adams S , Rugo HS , et al. Atezolizumab and nab‐paclitaxel in advanced triple‐negative breast cancer. New Engl J Med. 2018;379(22):2108‐2121. 30345906 10.1056/NEJMoa1809615

[24] Robson M , Im SA , Senkus E , et al. Olaparib for metastatic breast cancer in patients with a germline BRCA mutation. New Engl J Med. 2017;377(6):523‐533. 28578601 10.1056/NEJMoa1706450

