# Supplementary figures and images for: Biological subtype predicts locoregional recurrence after postmastectomy radiotherapy in Chinese breast cancer patients
Source: Cancer Med. 2020 Feb 12;9(7):2427–34. doi: 10.1002/cam4.2904 (PMC7131860; doi:10.1002/cam4.2904)

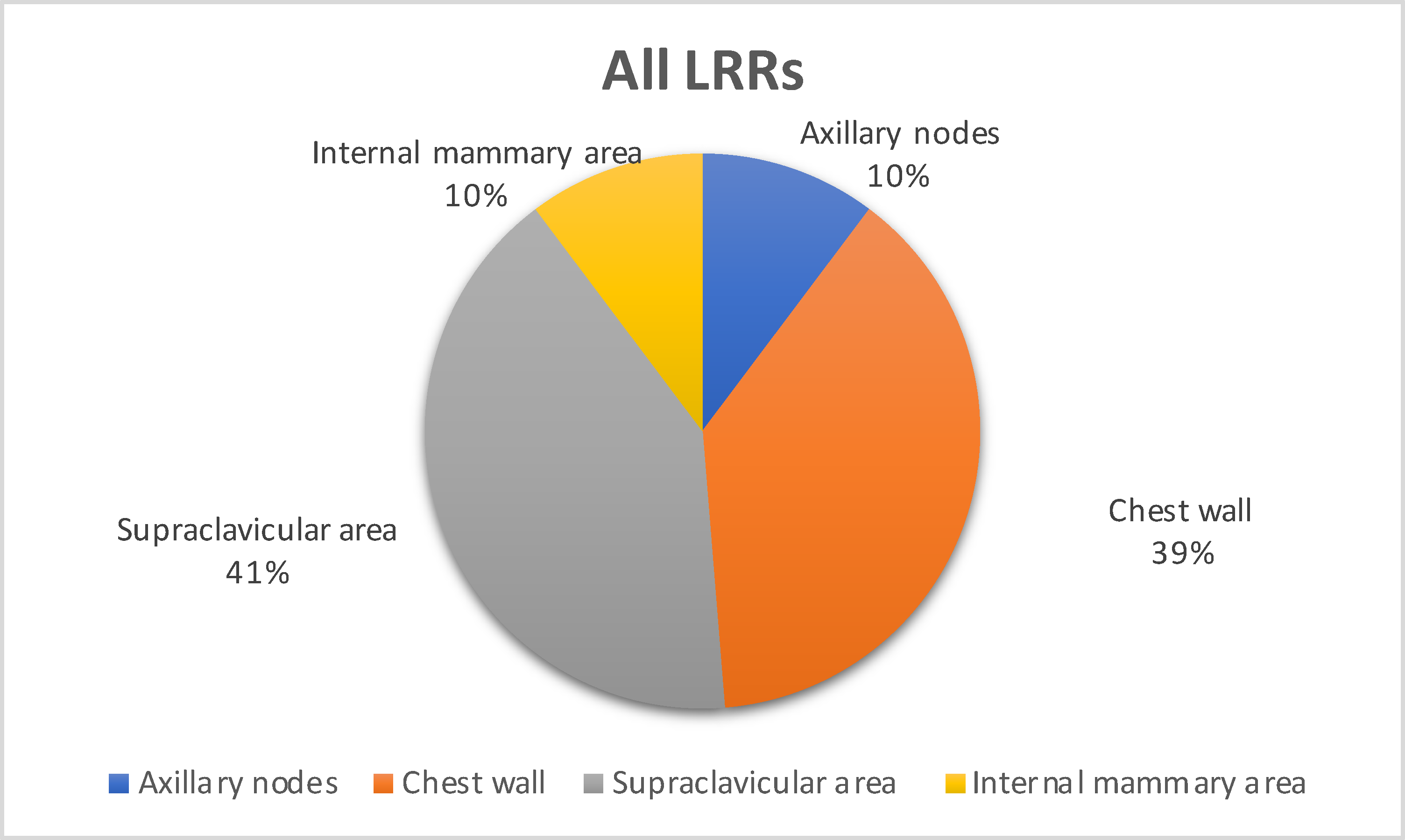

Supplement: Supplementary file 1 [file CAM4-9-2427-s001.tif]

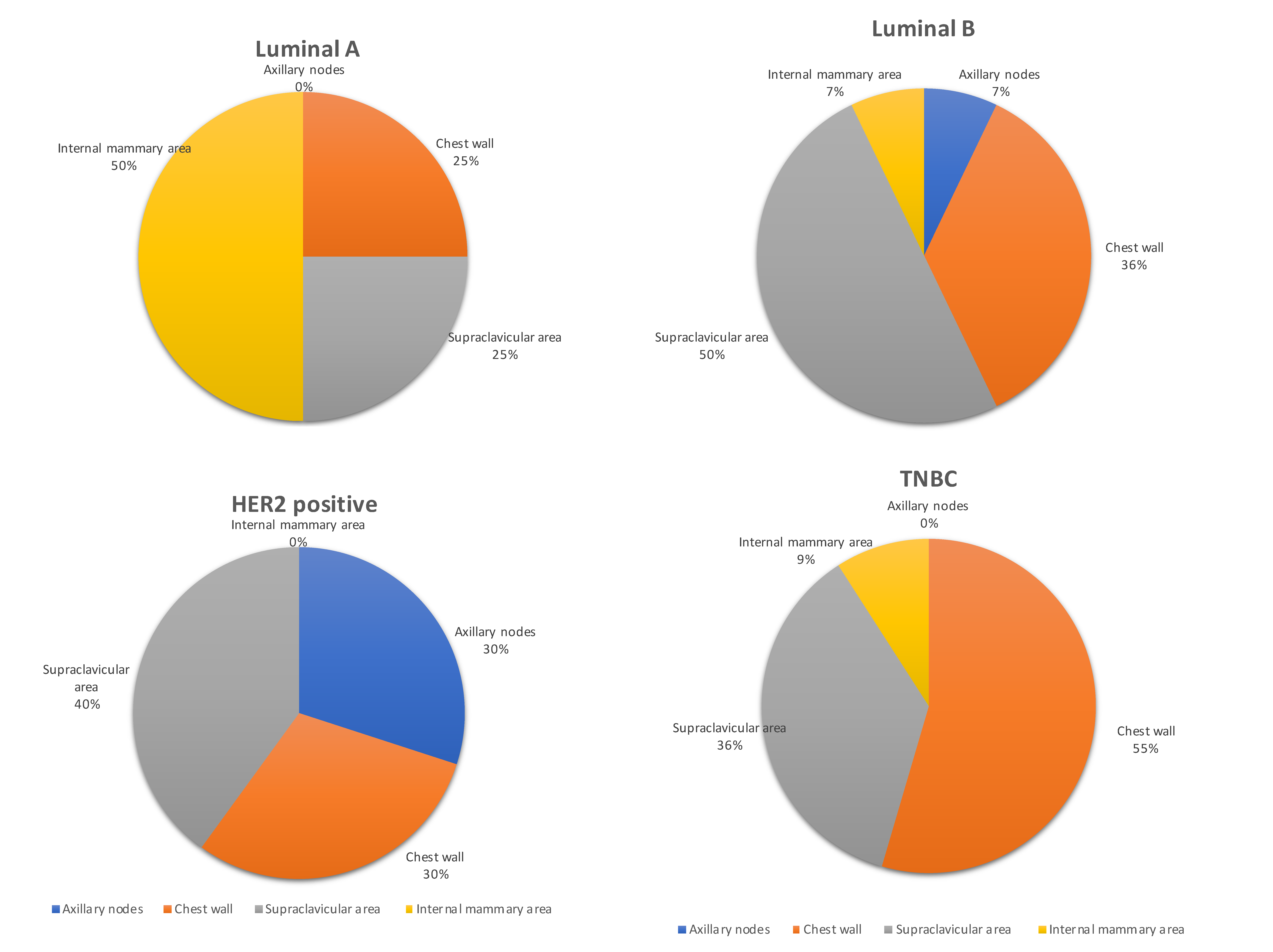

Supplement: Supplementary file 2 [file CAM4-9-2427-s002.tif]
